# Left Inferior Vena Cava and Right Retroaortic Renal Vein

**DOI:** 10.1155/2016/1270856

**Published:** 2016-02-03

**Authors:** Alberto Nania, Fabio Capilli, Eugenia Longo

**Affiliations:** ^1^Department of Radiology, NHS Lothian, Edinburgh EH16 4SA, UK; ^2^Department of Radiology, Dreifaltigkeits Hospital, 59555 Lippstadt, Germany; ^3^Department of Radiology, Azienda Ospedali Riuniti Papardo-Piemonte, 98123 Messina, Italy

## Abstract

Nowadays, incidental anatomical variants are frequent findings, due to the widespread diffusion of cross-sectional imaging. This case report illustrates a fairly uncommon anatomical variant, that is, the copresence of left inferior vena cava and retroaortic right renal vein reported in a 46-year-old lady, undergoing a staging CT for breast cancer. Although the patient was asymptomatic, the authors highlight potential risks related to the above-mentioned condition and the importance of correct identification and diagnosis of the findings.

## 1. Introduction

Incidental anatomical variants are commonly encountered in CT reporting. Due to its complex embryogenesis, the inferior vena cava (IVC) often presents several variations [1–3].

Despite being often asymptomatic, these represent a potential diagnostic pitfall for radiologist. Moreover, a detailed description of the anatomy of the IVC is particularly relevant for surgeons performing a presurgical assessment.

This paper presents a case review of an incidental finding of left IVC associated with retroaortic left inferior renal vein, in a female patient undergoing CT staging for breast carcinoma. In addition, the clinical significance of the variation is discussed.

## 2. Case Report

A 46-year-old lady underwent CT chest abdomen and pelvis for breast cancer staging. The CT was reported as free of secondary pathology; however, the below-discussed findings were observed.

**Figure 1:**
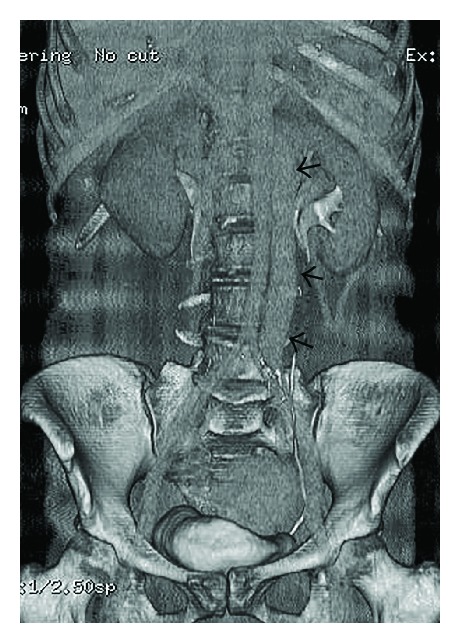
Left inferior vena cava (black arrows).

**Figure 2:**
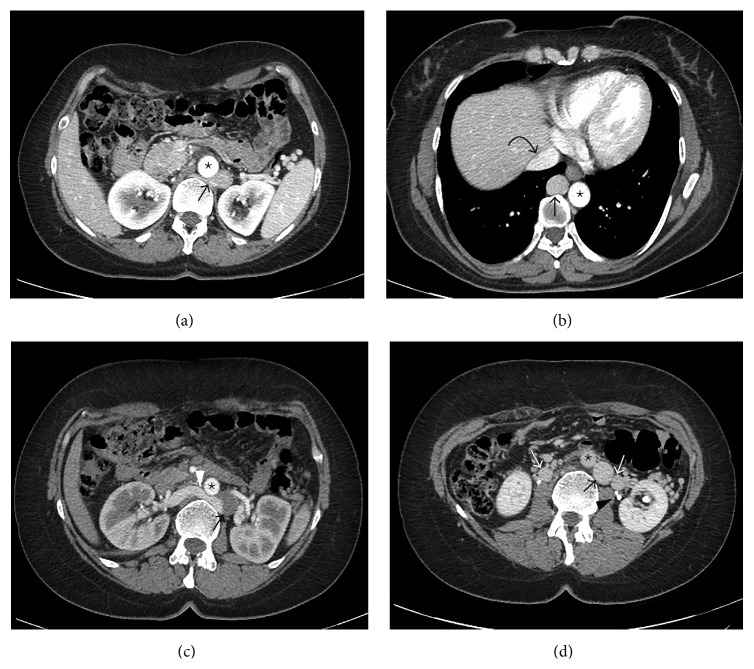
(a) Left IVC (black arrow) crosses the middle line behind the aorta (star). (b) Infrahepatic IVC drains into the Azygos vein (black straight arrow) that enters the thorax together with the aorta (star). Suprahepatic IVC (black curved arrow) is formed only from the hepatic veins and drains as usual into the right atrium. (c) Right renal vein (white arrowhead) crosses the middle line behind the aorta (star) to join the left IVC (black arrow). (d) Infrarenal IVC (black arrow) on the left side of the aorta (star). Large ovarian veins (white arrows) on both sides. Proximally duplicated left ureter (black arrowhead).

The CT scan shows the infrarenal tract of the IVC localised on the left side of the aorta. This left paramedian IVC is of normal calibre and crosses the middle line at the level of the celiac trunk, behind the aorta (Figure 1). At T 12, the IVC continues its way up to the chest as Azygos vein, passing through the aortic hiatus of the diaphragm. No embryologic remnants of the supracardinal veins are noted on the right side of the aorta. A large vein is located ventromedially to the ureter on both sides, consistent with large ovarian veins. Furthermore, the right renal vein follows an anomalous course, as it is situated posteriorly to the aorta to drain into the left-sided IVC. Finally, a partial duplication of the left ureter is noted (Figure 2).

## 3. Discussion

Embryogenesis of the IVC represents an intricate process that warrants a summary in order to better understand the aetiology of anatomical variations.

Between the 6th and 8th week of gestation, three pairs of embryonic vessels conjoin to form the infrahepatic IVC. In chronological order of appearance, they are named postcardinal, subcardinal, and supracardinal veins. It is important to remember that the process is indeed dynamic and involves formation and regression of the above-named pairs of embryologic vessels. A widely accepted scheme [4] divides the IVC development into three different parts: prerenal, renal, and infrarenal segments.

Under normal circumstances, the prerenal division of the IVC is formed following the union of the hepatic segment, which itself is a vitelline vein derivative, with the right subcardinal vein.

This process is preceded by anastomoses between the posterior cardinal veins, draining the blood flowing from the body wall caudal to the heart, and the subcardinal veins.

The postcardinal veins will atrophy after the anastomoses are established.

The renal segments of the IVC develop from a multiple anastomoses process that involves bilaterally all the three pairs of embryonic vessels.

Cranially, the supracardinal and subcardinal veins merge forming a circular structure that crosses the midline, dorsally to the aorta, named suprasubcardinal anastomosis.

Caudally, a similar process involves postcardinal and subcardinal veins, in pairs, forming the postsubcardinal anastomosis.

The renal segment of the IVC is formed by the confluence of these two systems, that is, suprasubcardinal and postsubcardinal anastomoses, named renal collar, which is followed by the atrophy of the posterior cardinal veins.

Finally, the infrarenal segment of the IVC seems to derive from the right supracardinal vein.

For further references, a comprehensive review of the embryogenesis of the IVC was published by Phillips [5].

A left IVC has a prevalence of 0.2%–0.5% [4, 5], resulting from regression of the right supracardinal vein with persistency of left supracardinal vein.

In the vast majority of cases, however, a left ICV is associated with a crossover of the vein anterior to the aorta, which therefore continues with a normally right-sided prerenal IVC.

This case associates the left IVC with the more common anomaly of retroaortic right renal vein. This has a prevalence of up to 2.1% as isolated abnormality [4].

With reference to the embryology, a retroaortic renal vein is found when the ventral arch of the renal collar goes into atrophy leaving the dorsal side of the arch patent.

Such a rare anatomical variation requires careful radiological assessment, as a misdiagnosis of lymphadenopathy represents a possible pitfall [6].

With regard to the retroaortic position of the renal vein, compression and entrapment of the latter between the aorta and the lumbar vertebrae have been observed, generating the so-called “posterior nutcracker syndrome.” This encompasses flank pain, haematuria, and proteinuria [7].

Furthermore, preoperative recognition of the anomaly is paramount both for open surgery and intravascular intervention [8], such as IVC filter positioning. In the latter, transjugular access may result in being technically difficult.

Several potential complications have been identified in light of IVC anatomical abnormalities.

Spontaneous rupture of abdominal aortic aneurism into left IVC has been described [9].

Moreover, the risk of deep venous thrombosis has been reported [10].

Some authors have described cases of acute DVT in young patients with abnormalities of IVC system, highlighting the possible need for anticoagulant therapy.

Specifically, up to 5% incidence of idiopathic DVT in young patients may be related to IVC anatomical variation [11], where the atypical crossover of the IVC from left to right side of the midline may lead to anomalous blood flow.

Finally, lower back pain may be correlated to IVC variations [7].

The contrast used in the case reported represented a valuable support towards the correct identification of the IVC; however, noncontrast CT may result in being more challenging.

## 4. Conclusion

We reported a case of left-sided IVC coexistent with retroaortic right renal vein, which, to the best of our knowledge, has not been described before in association.

This example of rare anatomical variation highlights the risks of misdiagnosis and potential complications correlated to abnormalities of IVC.

A thorough assessment and comprehensive knowledge of the possible anomalies are therefore necessary for correct diagnosis and management of such patients.

## References

[1] Moore K. L. (2000). *Clinically Oriented Anatomy*.

[2] Abrahams P. H., Craven J. L., Lumley J. S. P. (2005). *Illustrated Clinical Anatomy*.

[3] Sadler T. W. (2004). *Langman's Medical Embryology*.

[4] Bass J. E., Redwine M. D., Kramer L. A., Huynh P. T., Harris J. H. (2000). Spectrum of congenital anomalies of the inferior vena cava: cross-sectional imaging findings. *RadioGraphics*.

[5] Phillips E., Ferris E. J., Hipona F. A., Kahn P. C., Phillips E., Shapiro J. H. (1969). Embryology, normal anatomy, and anomalies. *Venography of the Inferior Vena Cava and Its Branches*.

[6] Siegfried M. S., Rochester D., Bernstein J. R., Miller J. W. (1983). Diagnosis of inferior vena cava anomalies by computerized tomography. *Computerized Radiology*.

[7] Spentzouris1 G., Zandian A., Cesmebasi A. (2014). The clinical anatomy of the inferior vena cava: a review of common congenital anomalies and considerations for clinicians. *Clinical Anatomy*.

[8] Rajakulasingam R., Francis R., Rajakulasingam R. (2013). Vena caval anomalies. *Journal of Clinical Imaging Science*.

[9] Davachi A. A. (1965). Acute spontaneous rupture of an arteriosclerotic aneurysm into an isolated left sided inferior vena cava. *American Journal of Cardiology*.

[10] Milani C., Constantinou M., Berz D., Butera J. N., Colvin G. A. (2008). Left sided inferior vena cava duplication and venous thromboembolism: case report and review of literature. *Journal of Hematology & Oncology*.

[11] Ongom P. A. (2013). A left inferior vena cava with crossover to the right: a case report. *International Journal of Anatomical Variations*.

